# Association of a rheumatoid arthritis susceptibility variant at the CCL21 locus with premature mortality in inflammatory polyarthritis patients

**DOI:** 10.1002/acr.20208

**Published:** 2010-05

**Authors:** Tracey M Farragher, Darren Plant, Edward Flynn, Steve Eyre, Diane Bunn, Wendy Thomson, Deborah Symmons, Anne Barton

**Affiliations:** 1 arc Epidemiology Unit, The University of ManchesterManchester, UK; 2 Norfolk Arthritis Register, University of East AngliaNorwich, UK

## Abstract

**Objective:**

To investigate whether recently identified rheumatoid arthritis (RA) susceptibility loci are also associated with disease severity, specifically all-cause and cardiovascular disease (CVD) mortality, in patients with inflammatory polyarthritis (IP).

**Methods:**

Subjects with recent-onset IP were recruited from the Norfolk Arthritis Register. Seventeen RA susceptibility single-nucleotide polymorphisms (SNPs) were tested using Sequenom MassArray iPLEX chemistry. Vital status was ascertained from central records. The association of SNP allele carriage with mortality risk was assessed using Cox proportional hazards models after adjusting by sex. The mortality risks of those SNP alleles found to be associated were then stratified by baseline anti–citrullinated peptide (anti-CCP) antibody and shared epitope (SE) status.

**Results:**

All SNPs were successfully genotyped in 2,324 IP subjects. The presence of 2 copies of the risk allele rs2812378 mapping to the *CCL21* gene predicted all-cause mortality (hazard ratio [HR] 1.40, 95% confidence interval [95% CI] 1.04–1.87), whereas risk allele carriage also predicted increased CVD mortality (HR 1.33, 95% CI 1.01–1.75). The highest mortality risks were seen in anti-CCP antibody–positive subjects with 2 copies of the *CCL21* risk alleles and 2 copies of the SE (all-cause HR 3.20, 95% CI 1.52–6.72; CVD HR 3.73, 95% CI 1.30–10.72).

**Conclusion:**

In this large study, we found that carriage of *CCL21* risk alleles was associated with premature mortality in IP independently of anti-CCP antibody and SE status. Interestingly, CCL21 expression has been reported in atherosclerotic plaques supporting the thesis that the increased CVD mortality in IP patients may be mediated by shared inflammatory mechanisms.

## INTRODUCTION

With better long-term control of disease activity now possible in patients with rheumatoid arthritis (RA) due to the earlier, more aggressive use of disease-modifying antirheumatic drugs and the advent of biologic therapies, interest is now focusing on preventing the comorbidities that occur more commonly in this condition. Patients with RA die prematurely, often from cardiovascular disease (CVD) (1). However, traditional CVD risk factors, such as smoking and dyslipidemia, do not appear to account for all of the excess risk (2). Because there is evidence for an inflammatory component in both RA and CVD, we hypothesized that shared genetic risk factors may account for the excess prevalence of CVD in RA.

We have previously reported that the major RA susceptibility locus, the group of HLA–DRB1 alleles collectively referred to as the shared epitope (SE), is associated with both all-cause and CVD mortality in patients with inflammatory polyarthritis (IP) and RA, but that the second-strongest susceptibility gene, *PTPN22*, shows no such association (3). Over the last 2 years, there has been considerable progress in identifying further RA susceptibility genes, and at least 11 other loci have been reported to be associated with RA in more than one well-powered study (4–12). Many of these susceptibility genes are involved in immune regulation and inflammation, and many show overlap with other complex immune/inflammatory diseases. For example, single-nucleotide polymorphism (SNP) markers mapping to a region on chromosome 9 encompassing the *TRAF1* and part of the *C5* genes, which have been widely confirmed as being associated with RA susceptibility, were reported to be associated with CVD mortality in a small, hospital-based series of RA patients (13). The aim of the current study was to investigate whether the recently identified RA susceptibility loci are also associated with all-cause mortality, and specifically CVD mortality, in an inception cohort of patients with IP followed prospectively.

## PATIENTS AND METHODS

### Study design

A cohort analysis was undertaken comparing survival in subjects with IP according to RA susceptibility SNP marker genotype.

### Subjects

Subjects were recruited from the Norfolk Arthritis Register (NOAR), a primary care–based inception cohort of subjects with recent-onset IP that has been described previously (14). Briefly, from 1989 onward, the NOAR aimed to recruit all adults age ≥16 years who had swelling of at least 2 joints persisting for at least 4 weeks. The NOAR covers the former Norwich Health Authority with notification of cases via general practitioners or attendance at hospitals within the catchment area (14). Those who were subsequently diagnosed by a hospital consultant as having a condition other than RA, IP, psoriatic arthritis, or postviral arthritis were excluded. Between 1989 and 2005, 2,469 consecutive subjects who satisfied the above criteria and had a DNA sample available for genotyping were referred to the NOAR. All of the subjects were white.

### Data collection

A research nurse conducted a structured interview and clinical examination at baseline. Demographic data collected included the age at symptom onset, sex, smoking status, and time from symptom onset to presentation to the NOAR. A blood sample was tested for rheumatoid factor (RF), anti–cyclic citrullinated peptide (anti-CCP) antibody, and C-reactive protein level. RF was measured using a latex agglutination technique, where a titer of ≥1:40 was classified as RF positive. Anti-CCP antibodies were detected using the Axis-Shield DIASTAT kit according to the manufacturer's instructions, where a concentration of >5 units/ml was classified as anti-CCP antibody positive (Axis-Shield). The American College of Rheumatology (ACR; formerly the American Rheumatism Association) 1987 criteria (15) for the classification of RA were applied at baseline.

### Genotyping

Seventeen SNPs were selected for genotyping and tested using Sequenom MassArray iPLEX chemistry according to the manufacturer's instructions (online at: http://www.sequenom.com). The SNPs were markers associated with RA susceptibility in cohorts tested by our group and others, and consisted of: rs1160542 (*AFF3*); rs2812378 (*CCL21*); rs763361 (*CD226*); rs4810485 (*CD40*); rs3087243 and rs231775 (*CTLA4*); rs6822844 (*IL2/IL21*); rs2104286 (*IL2RA*); rs743777 (*IL2RB*); rs6897932 (*IL7R*); rs1678542 (*KIF5A*); rs7574865 (*STAT4*); rs13207033, rs5029937, and rs6920220 (*OILG3/TNFAIP3*); and rs10760130 and rs2900180 (*TRAF1/C5*). Only those SNPs with a genotype success rate of >85% were included in the analysis. HLA genotyping was performed as described previously (16).

### Ascertainment of deaths

Death was ascertained by record linkage with the UK Office for National Statistics (ONS). The cause of death was coded using the International Statistical Classification of Diseases and Related Health Problems, Tenth Revision (ICD-10) (17). Cause of death was grouped by ICD-10 chapter. Patients were followed from disease symptom onset until death or December 31, 2007, whichever was sooner. The ONS notified the NOAR if a patient moved from the UK or was no longer registered with a general practitioner, giving a date of embarkation. For these subjects, followup was censored to the time of embarkation.

### Statistical analysis

The association of SNP marker allele carriage with mortality risk was assessed using Cox proportional hazards models after adjusting by sex. Each subject entered the analysis on their date of birth and became at risk of mortality on the date of symptom onset. Analyses were undertaken for all-cause and CVD mortality, which we defined as any mention of CVD (ICD-10 codes I00–I99) on the death certificate. This analysis was carried out on the IP cohort and the subgroup that satisfied the ACR criteria for RA by the end of 2007 or death, whichever was sooner.

Because smoking is a known risk factor for CVD, analysis of the SNPs with evidence for association with all-cause or CVD mortality was repeated after adjusting for baseline smoking status (current/ever/never) to determine whether smoking was an important confounding factor. We have previously found anti-CCP antibody status, a marker of disease severity, and carriage of 2 copies of SE alleles to be predictors of mortality (3). For any SNP associated with mortality in the current study, interaction analysis with SE status (0/1 versus 2 SE alleles) and anti-CCP antibody status was assessed via Cox proportional hazards models after adjusting by sex. All analyses were undertaken using Stata, version 9.2 (StataCorp).

## RESULTS

After applying strict quality control measures, all 17 SNPs were successfully genotyped in 2,324 IP subjects. Of the 2,324 IP subjects, 1,027 (44.2%) satisfied the ACR criteria for RA at baseline (Table 1). By December 31, 2007, 399 subjects (17.2%) had died and CVD was recorded on the death certificate for 216 subjects (54% of all deaths).

**Table 1:** Clinical and demographic characteristics of the entire IP cohort*

	IP cohort (n = 2,324)
Age at symptom onset, median (IQR) years	54 (42–66)
Delay to registration, median (IQR) months	7 (3–14)
CRP level (n = 1,985), median (IQR) mg/dl	7.7 (1.5–18.3)
Women	1,537 (66.1)
Satisfy ACR criteria for RA at baseline	1,027 (44.2)
RF positive at baseline (n = 2,169)†	690 (31.8)
Anti-CCP antibody positive at baseline (n = 2,046)	624 (30.5)
Copies of SE alleles (n = 1,567)	
0/1	1,373 (87.6)
2	194 (12.4)
Smoking status at baseline (n = 2,010)	
Never smoked	524 (26.1)
Ex-smoker	911 (45.3)
Current smoker	575 (28.6)
Died by the end of 2007	399 (17.2)
Died (CVD) by the end of 2007	229 (9.9)

* Values are the number (percentage) unless otherwise indicated. IP = inflammatory polyarthritis; IQR = interquartile range; CRP = C-reactive protein; ACR = American College of Rheumatology; RA = rheumatoid arthritis; RF = rheumatoid factor; anti-CCP = anti–cyclic citrullinated peptide; SE = shared epitope; CVD = cardiovascular disease.

† Titer ≥1:40.

### RA susceptibility alleles as predictors of all-cause and CVD mortality

In order to confirm that the data set used in the current analysis was comparable with RA cohorts tested previously to investigate susceptibility loci, we first compared allele frequencies in the IP subjects with 875 UK controls for whom genotyping data were available for the same SNPs. The odds ratios in the current IP cohort were similar (same direction and magnitude) to those found in the larger RA case–control cohorts reported previously, although as expected due to the smaller sample size, these associations were only statistically significant for 3 of the variants (Supplementary Table 1, available in the online version of this article at http://www3.interscience.wiley.com/journal/77005015/home).

Only 1 SNP was associated with all-cause mortality (Table 2) in the IP cohort. Carriage of 2 copies of the risk allele of rs2812378 mapping to the *CCL21* gene was associated with a significantly increased risk of all-cause mortality compared with carriage of 2 copies of the non–risk allele (hazard ratio [HR] adjusted by sex 1.40, 95% confidence interval [95% CI] 1.04–1.87). Carriage of this risk allele was also associated with CVD mortality compared with those who were homozygous for the non–risk allele (HR adjusted by sex 1.33, 95% CI 1.01–1.75) (Table 3). No association was found with either all-cause or CVD mortality and carriage of the risk alleles for any other loci (Supplementary Tables 2 and 3, available in the online version of this article at http://www3.interscience.wiley.com/journal/77005015/home), including SNPs rs10760130 and rs2900180 mapping to the *TRAF1/C5* locus, which were previously reported to be associated with CVD mortality (Tables 2 and 3). No statistically significant associations were found for either all-cause or CVD mortality with the RA susceptibility alleles in the RA subgroup, although the estimates for the mortality risk are similar to those from the entire IP cohort.

**Table 2:** All-cause mortality at the end of 2007 by selected RA susceptibility SNP loci*

	No.	Unadjusted, HR (95% CI)	Adjusted by sex, HR (95% CI)
Inflammatory polyarthritis			
rs2900180			
0 risk alleles	902	1.0	1.0
1 risk allele	1,052	1.09 (0.88–1.36)	1.1 (0.88–1.36)
1/2 risk alleles		1.06 (0.87–1.31)	1.07 (0.87–1.31)
2 risk alleles	343	0.98 (0.73–1.34)	0.99 (0.73–1.34)
rs10760130			
0 risk alleles	673	1.0	1.0
1 risk allele	1,118	0.98 (0.77–1.23)	0.98 (0.78–1.24)
1/2 risk alleles		1.01 (0.81–1.26)	1.02 (0.82–1.27)
2 risk alleles	510	1.10 (0.83–1.44)	1.10 (0.84–1.45)
rs2812378			
0 risk alleles	954	1.0	1.0
1 risk allele	1,072	0.97 (0.78–1.20)	0.98 (0.79–1.22)
1/2 risk alleles		1.05 (0.86–1.29)	1.07 (0.87–1.30)
2 risk alleles	274	1.37 (1.03–1.84)†	1.40 (1.04–1.87)†
RA subgroup (met ACR criteria for RA by the end of 2007)			
rs2900180			
0 risk alleles	609	1.0	1.0
1 risk allele	731	1.24 (0.96–1.60)	1.25 (0.97–1.61)
1/2 risk alleles		1.18 (0.93–1.51)	1.19 (0.93–1.51)
2 risk alleles	236	1.01 (0.70–1.45)	1.01 (0.70–1.45)
rs10760130			
0 risk alleles	454	1.0	1.0
1 risk allele	780	1.07 (0.81–1.40)	1.07 (0.81–1.41)
1/2 risk alleles		1.06 (0.82–1.38)	1.07 (0.82–1.38)
2 risk alleles	344	1.05 (0.76–1.47)	1.06 (0.76–1.48)
rs2812378			
0 risk alleles	661	1.0	1.0
1 risk allele	725	0.94 (0.73–1.21)	0.94 (0.73–1.22)
1/2 risk alleles		0.99 (0.79–1.26)	1.00 (0.79–1.27)
2 risk alleles	191	1.18 (0.84–1.67)	1.20 (0.85–1.69)

* RA = rheumatoid arthritis; SNP = single-nucleotide polymorphism; HR = hazard ratio; 95% CI = 95% confidence interval; ACR = American College of Rheumatology.

† *P* < 0.05.

**Table 3:** CVD mortality at the end of 2007 by selected RA susceptibility SNP loci*

	No.	Unadjusted, HR (95% CI)	Adjusted by sex, HR (95% CI)
Inflammatory polyarthritis			
rs2900180			
0 risk alleles	902	1.0	1.0
1 risk allele	1,052	1.14 (0.86–1.51)	1.14 (0.86–1.52)
1/2 risk alleles		1.05 (0.80–1.37)	1.05 (0.80–1.38)
2 risk alleles	343	0.79 (0.51–1.21)	0.80 (0.52–1.23)
rs10760130			
0 risk alleles	673	1.0	1.0
1 risk allele	1,118	0.99 (0.73–1.33)	0.99 (0.74–1.35)
1/2 risk alleles		0.95 (0.72–1.27)	0.96 (0.72–1.28)
2 risk alleles	510	0.88 (0.60–1.28)	0.89 (0.61–1.30)
rs2812378			
0 risk alleles	954	1.0	1.0
1 risk allele	1,072	1.23 (0.92–1.64)	1.25 (0.93–1.67)
1/2 risk alleles		1.31 (0.99–1.72)	1.33 (1.01–1.75)†
2 risk alleles	274	1.60 (1.08–2.35)†	1.63 (1.11–2.41)†
RA subgroup (met ACR criteria for RA by the end of 2007)			
rs2900180			
0 risk alleles	609	1.0	1.0
1 risk allele	731	1.26 (0.91–1.74)	1.28 (0.92–1.76)
1/2 risk alleles		1.10 (0.81–1.51)	1.12 (0.82–1.53)
2 risk alleles	236	0.67 (0.39–1.15)	0.68 (0.40–1.16)
rs10760130			
0 risk alleles	454	1.0	1.0
1 risk allele	780	1.08 (0.77–1.53)	1.09 (0.77–1.54)
1/2 risk alleles		0.97 (0.70–1.36)	0.99 (0.71–1.38)
2 risk alleles	344	0.74 (0.46–1.17)	0.76 (0.48–1.20)
rs2812378			
0 risk alleles	661	1.0	1.0
1 risk allele	725	1.08 (0.78–1.51)	1.11 (0.80–1.55)
1/2 risk alleles		1.15 (0.84–1.56)	1.18 (0.87–1.61)
2 risk alleles	191	1.35 (0.87–2.10)	1.41 (0.91–2.19)

* CVD = cardiovascular disease; RA = rheumatoid arthritis; SNP = single-nucleotide polymorphism; HR = hazard ratio; 95% CI = 95% confidence interval; ACR = American College of Rheumatology.

† *P* < 0.05.

To explore whether the increased mortality risk was as a result of traditional CVD risk factors, we adjusted the HRs in Tables 2 and 3 by smoking status at baseline (never, previous, current). We found very little change in the estimates for mortality risk in the selected susceptibility SNP loci. In particular, carriage of 2 copies of the risk allele rs2812378 mapping to the *CCL21* gene remained associated with a significantly increased risk of all-cause mortality (HR adjusted by sex and smoking 1.38, 95% CI 1.02–1.87) and CVD mortality (HR adjusted by sex and smoking 1.66, 95% CI 1.11–2.48) compared with those who were homozygous for the non–risk allele. Furthermore, carriage of this risk allele remained associated with CVD mortality compared with those who were homozygous for the non–risk allele (HR adjusted by sex and smoking 1.37, 95% CI 1.03–1.81). No association was found with either all-cause or CVD mortality or carriage of the risk alleles for any other loci after adjustment for smoking and sex.

### *CCL21* risk allele as a predictor of mortality by baseline anti-CCP antibody status

Carriage of 2 copies of the *CCL21* risk allele was associated with increased all-cause mortality in patients who were anti-CCP antibody negative (HR 1.48, 95% CI 1.00–2.20), but was stronger in those who were anti-CCP antibody positive (HR for 2 copies versus 0 copies of risk allele 1.97, 95% CI 1.21–3.21) (Table 4). Increased risk of CVD mortality and carriage of the *CCL21* risk allele was also seen in those who were anti-CCP antibody negative, although it did not reach statistical significance (HR for 1 or 2 copies versus 0 copies 1.45, 95% CI 0.99–2.12), and was higher in those who were anti-CCP antibody positive (HR 2.36, 95% CI 1.54–3.61). Stratification analysis of the other RA susceptibility alleles by anti-CCP antibody status revealed no additional mortality risk in anti-CCP antibody–positive or –negative subgroups (data not shown).

**Table 4:** All-cause and CVD mortality (adjusted by sex) by RA susceptibility, SNP locus, and anti-CCP antibody status*

	All-cause mortality/anti-CCP antibody negative		All-cause mortality/anti-CCP antibody positive		CVD mortality/anti-CCP antibody negative		CVD mortality/anti-CCP antibody positive	
	No.	HR (95% CI)	No.	HR (95% CI)	No.	HR (95% CI)	No.	HR (95% CI)
rs2812378								
0 risk alleles	583	1.0	262	1.52 (1.07–2.15)	583	1.0	262	2.18 (1.36–3.49)
1 risk allele	664	0.96 (0.72–1.29)	278	1.51 (1.08–2.13)	664	1.38 (0.93–2.06)	278	2.27 (1.44–3.58)
2 risk alleles	159	1.48 (1.00–2.20)	78	1.97 (1.21–3.21)	159	1.72 (0.98–3.01)	78	2.70 (1.41–5.16)
1/2 risk alleles	823	1.06 (0.81–1.39)	356	1.62 (1.18–2.21)	823	1.45 (0.99–2.12)	356	2.36 (1.54–3.61)

* CVD = cardiovascular disease; RA = rheumatoid arthritis; SNP = single-nucleotide polymorphism; anti-CCP = anti–cyclic citrullinated peptide; HR = hazard ratio; 95% CI = 95% confidence interval.

### *CCL21* risk allele as a predictor of mortality by SE status

The association of carriage of the *CCL21* risk allele with increased CVD mortality was independent of SE status (HR in those with 0 or 1 copy of the SE 1.44, 95% CI 1.03–2.03; HR in those with 2 copies of the SE 1.87, 95% CI 1.14–3.06) (Table 5). The increased all-cause mortality risk in those with 2 copies of the *CCL21* risk allele was only seen in those with 2 copies of the SE (HR 2.67, 95% CI 1.43–5.00).

**Table 5:** All-cause and CVD mortality (adjusted by sex) by RA susceptibility, SNP locus, and SE*

	All-cause mortality/0/1 copy of SE alleles		All-cause mortality/2 copies of SE alleles		CVD mortality/0/1 copy of SE alleles		CVD mortality/2 copies of SE alleles	
	No.	HR (95% CI)	No.	HR (95% CI)	No.	HR (95% CI)	No.	HR (95% CI)
rs2812378								
0 risk alleles	613	1.0	98	1.54 (0.95–2.50)	613	1.0	98	1.69 (0.85–3.36)
1 risk allele	695	1.08 (0.83–1.41)	128	0.98 (0.62–1.57)	695	1.43 (1.00–2.05)	128	1.63 (0.94–2.83)
2 risk alleles	178	1.24 (0.86–1.79)	36	2.67 (1.43–5.00)	178	1.50 (0.91–2.47)	36	3.21 (1.37–7.54)
1/2 risk alleles	873	1.12 (0.87–1.43)	164	1.26 (0.84–1.87)	873	1.44 (1.03–2.03)	164	1.87 (1.14–3.06)

* CVD = cardiovascular disease; RA = rheumatoid arthritis; SNP = single-nucleotide polymorphism; SE = shared epitope; HR = hazard ratio; 95% CI = 95% confidence interval.

### Interaction of SE, anti-CCP antibody, and *CCL21* risk allele as predictors of mortality

Using a multiplicative model, subjects with 2 copies of the *CCL21* risk allele and 2 copies of the SE and who were anti-CCP antibody positive had a significantly higher all-cause mortality (HR 3.20, 95% CI 1.52–6.72) and CVD mortality (HR 3.73 95% CI 1.30–10.72) when compared with those who were anti-CCP antibody negative who had 0 or 1 copy of the SE and 0 copies of the *CCL21* risk allele (Table 6). When using an additive model to estimate the mortality associated with the combination of these 3 risk factors, we found lower HRs (all-cause HRs 1.99, 1.20, and 3.32; CVD HRs 2.98, 1.53, and 5.80). This indicates that there is an interaction between the 2 genetic markers and anti-CCP antibody status rather than just an additive effect on the mortality risk.

**Table 6:** All-cause and CVD mortality (adjusted by sex) at the end of 2007 by RA susceptibility, SNP locus, SE, and anti-CCP antibody status*

	All-cause mortality/0/1 copy of SE alleles		All-cause mortality/2 copies of SE alleles		CVD mortality/0/1 copy of SE alleles		CVD mortality/2 copies of SE alleles	
	No.	HR (95% CI)	No.	HR (95% CI)	No.	HR (95% CI)	No.	HR (95% CI)
Anti-CCP antibody negative								
rs2812378								
0 risk alleles	387	1.0	28	1.62 (0.59–4.49)	387	1.0	28	–†
1 risk allele	430	1.10 (0.78–1.54)	48	0.59 (0.24–1.47)	430	1.56 (0.98–2.49)	48	0.97 (0.34–2.75)
2 risk alleles	112	1.39 (0.87–2.22)	7	1.06 (0.15–7.71)	112	1.50 (0.76–2.95)	7	2.43 (0.33–17.98)
Anti-CCP antibody positive								
rs2812378								
0 risk alleles	154	1.47 (0.95–2.29)	56	1.90 (1.04–3.48)	154	1.91 (1.04–3.49)	56	3.15 (1.48–6.72)
1 risk allele	178	1.63 (1.08–2.45)	56	1.34 (0.69–2.62)	178	2.02 (1.15–3.57)	56	3.50 (1.78–6.89)
2 risk alleles	41	1.40 (0.67–2.93)	24	3.20 (1.52–6.72)	41	1.90 (0.73–4.92)	24	3.73 (1.30–10.72)
Anti-CCP antibody negative								
rs2812378								
0 risk alleles	387	1.0	28	1.62 (0.59–4.49)	387	1.0	28	–†
1/2 risk alleles	542	1.16 (0.84–1.60)	55	0.64 (0.28–1.48)	542	1.55 (0.99–2.42)	55	1.10 (0.42–2.84)
Anti-CCP antibody positive								
rs2812378								
0 risk alleles	154	1.47 (0.95–2.28)	56	1.90 (1.04–3.47)	154	1.90 (1.04–3.49)	56	3.14 (1.47–6.70)
1/2 risk alleles	219	1.58 (1.08–2.33)	80	1.80 (1.06–3.06)	219	2.00 (1.17–3.41)	80	3.55 (1.92–6.57)

* CVD = cardiovascular disease; RA = rheumatoid arthritis; SNP = single-nucleotide polymorphism; SE = shared epitope; anti-CCP = anti–cyclic citrullinated peptide; HR = hazard ratio; 95% CI = 95% confidence interval.

† Too few events to calculate robust estimates.

## DISCUSSION

To our knowledge, this is the first study to explore the association of those SNPs that have recently been identified as RA susceptibility loci with all-cause and CVD mortality in subjects with IP. Carriage of 1 or more risk alleles of rs2812378 mapping to the *CCL21* gene was associated with increased CVD mortality, whereas carriage of 2 copies of the risk allele was associated with increased all-cause mortality. These effects on mortality were independent of anti-CCP antibody and SE status.

The *CCL21* gene is one of several Cys-Cys cytokine genes clustered on the short arm of chromosome 9. The CCL21 protein is a chemokine that is involved in homing lymphocytes to secondary lymphoid organs. Expression of this chemokine is associated with ectopic lymphoid structures and has been implicated in the organization of lymphoid tissue affected by RA (18). Furthermore, the CCL21 molecule and its receptor CCR7 have a possible role in the development of atherosclerosis by recruiting T cells and macrophages to the atherosclerotic lesions and by promoting inflammatory responses in these cells (19,20). The association between the risk allele at this gene and mortality, particularly CVD mortality, in RA highlights the potential that the excess CVD mortality observed in patients with RA may be related to abnormalities in the inflammatory process (1).

The size of the cohort gave us the potential to explore the effect of a combination of genetic predictors on mortality. The failure of SE status (0/1 versus 2) to explain the elevated CVD mortality risk of patients with risk alleles at the *CCL21* locus may indicate an independent effect to that which we have previously found for the SE (3). We were unable to replicate the recently found association between SNP markers mapping to *TRAF1/C5* and CVD mortality (13). Because we had more than 90% power to detect statistically significant effects, for the mortality risks estimated for the 2 *TRAF1/C5* SNPs, rs10760130 and rs2900180, from the previous report, our results are unlikely to represent false-negative findings. However, there are differences in the types of patients studied (hospital RA rather than primary care–based IP), which may explain the lack of association that we observed.

Because this study used a primary care–based cohort of subjects with IP, it had limited selection bias. Furthermore, the sample size was large and subjects had long followup, and therefore we were able to produce robust estimates of the influence of selected SNP markers on mortality. However, we cannot rule out the possibility of a false-positive finding at the *CCL21* locus because no adjustment was made for multiple testing. The finding will need to be replicated in other cohorts before the SNP can be considered as a confirmed predictor of all-cause and CVD mortality.

A potential limitation of the current study could be the exclusion of subjects from the analysis, either because they did not provide a DNA sample (n = 1,264) or were lost to followup by the ONS (n = 23), if these individuals were systematically different from the subjects included in the study. However, no systematic differences in clinical characteristics were detected (data not shown). The initial analysis was undertaken in the IP cohort as a whole and in the subgroup satisfying the ACR criteria for RA. We did not find significant associations with any of the RA susceptibility alleles and mortality in the RA subgroup. However, there was little difference in the estimates for the RA subgroup compared with the entire IP cohort, and therefore the lack of significant findings could be a result of the smaller sample size. It might be argued that we should have also investigated the mortality risk for the RA susceptibility alleles in the RF-positive individuals, particularly because we have previously found that RF positivity is a predictor of mortality (3,21). However, in this cohort, we found that anti-CCP antibodies were a stronger predictor of mortality than RF. Compared with those negative for both RF and anti-CCP antibodies, those anti-CCP antibody positive and RF negative had an increased mortality risk (all-cause HR 2.24, 95% CI 1.46–3.43), whereas those RF positive and anti-CCP antibody negative did not have an increased mortality risk (all-cause HR 0.90, 95% CI 0.50–1.60).

Although we have found evidence that a marker associated with RA susceptibility may contribute to CVD mortality, the combination of anti-CCP antibodies, *CCL21*, and SE status does not explain all of the increased risk. Furthermore, even with the large sample size, we had relatively few cases with the combination of the 3 risk factors. A well-powered, unbiased, genome-wide association study has greater potential to identify further markers of CVD in RA compared with candidate gene studies, and may be required to find additional genetic risk factors that predict outcome. Identification of such markers would be an important clinical advance because it could allow better targeting of preventative therapies (e.g., lifestyle modification, aggressive treatment) to those patients with IP who are at high risk of premature mortality from CVD.

## AUTHOR CONTRIBUTIONS

All authors were involved in drafting the article or revising it critically for important intellectual content, and all authors approved the final version to be submitted for publication. Dr. Barton had full access to all of the data in the study and takes responsibility for the integrity of the data and the accuracy of the data analysis.

**Study conception and design.** Farragher, Plant, Flynn, Thomson, Symmons, Barton.

**Acquisition of data.** Plant, Flynn, Eyre, Bunn, Symmons.

**Analysis and interpretation of data.** Farragher, Thomson.
