# Supplementary material for: Association of a rheumatoid arthritis susceptibility variant at the CCL21 locus with premature mortality in inflammatory polyarthritis patients
Source: Arthritis Care Res (Hoboken). 2010 May;62(5):676–82. doi: 10.1002/acr.20208 (PMC2936115; doi:10.1002/acr.20208)
Supplement: Supplementary file 1 [file acr0062-0676-SD1.doc]

**Supplementary Table 1.** Association with RA susceptibility

|  |  | **IP** | | | | **RA subgroup** | | | |
| --- | --- | --- | --- | --- | --- | --- | --- | --- | --- |
|  | H-W control | OR | 95% CI | | p value | OR | 95% CI | | p value |
| **rs2104286** | 0.42 | 0.94 | 0.83 | 1.07 | 0.35 | 0.93 | 0.81 | 1.06 | 0.29 |
| **rs1678542** | 0.42 | 0.87 | 0.77 | 0.97 | 0.02 | 0.86 | 0.77 | 0.98 | 0.02 |
| **rs763361** | 0.62 | 1.06 | 0.95 | 1.19 | 0.29 | 1.06 | 0.94 | 1.19 | 0.35 |
| **rs3087243** | 0.29 | 0.96 | 0.86 | 1.07 | 0.47 | 0.96 | 0.86 | 1.09 | 0.54 |
| **rs1160542** | 0.62 | 1.21 | 1.08 | 1.35 | 0.001 | 1.24 | 1.10 | 1.39 | 0.001 |
| **rs6822844** | 0.91 | 0.84 | 0.73 | 0.97 | 0.02 | 0.81 | 0.69 | 0.94 | 0.01 |
| **rs6920220** | 0.03 | 1.11 | 0.97 | 1.27 | 0.13 | 1.10 | 0.95 | 1.26 | 0.20 |
| **rs2900180** | 0.22 | 1.11 | 0.99 | 1.25 | 0.09 | 1.12 | 0.99 | 1.27 | 0.07 |
| **rs10760130** | 0.62 | 1.08 | 0.96 | 1.21 | 0.19 | 1.08 | 0.96 | 1.21 | 0.23 |
| **rs2812378** | 0.08 | 1.07 | 0.95 | 1.20 | 0.28 | 1.07 | 0.94 | 1.21 | 0.31 |
| **rs231775** | 0.94 | 1.00 | 0.89 | 1.12 | 0.98 | 0.98 | 0.87 | 1.11 | 0.76 |
| **rs7574865** | 0.77 | 1.01 | 0.89 | 1.16 | 0.86 | 1.06 | 0.92 | 1.22 | 0.45 |
| **rs4810485** | 1.00 | 0.95 | 0.83 | 1.08 | 0.41 | 0.92 | 0.80 | 1.06 | 0.24 |
| **rs743777** | 0.24 | 1.05 | 0.81 | 1.36 | 0.73 | 1.11 | 0.84 | 1.45 | 0.46 |
| **rs6897932** | 0.50 | 0.89 | 0.79 | 1.01 | 0.08 | 0.89 | 0.78 | 1.02 | 0.10 |
| **rs5029937** | 0.57 | 1.15 | 0.84 | 1.58 | 0.43 | 1.18 | 0.84 | 1.64 | 0.37 |
| **rs13207033** | 0.36 | 0.96 | 0.84 | 1.09 | 0.53 | 0.97 | 0.84 | 1.11 | 0.65 |

**Supplementary Table 2.** All-cause mortality by RA susceptibility SNP loci

| **SNP and no. of copies of risk allele** | | **All cause Mortality as at end of 2007, adjusted by sex** | | | | | | | | | | | |
| --- | --- | --- | --- | --- | --- | --- | --- | --- | --- | --- | --- | --- | --- |
|  |  | **IP cohort** | | | | | | **RA subgroup  (met ACR criteria for RA by end of 2007)** | | | | | |
|  |  | **n** | **HR** | **(95 %CI)** |  | **HR** | **(95 %CI)** | **n** | **HR** | **(95 %CI)** |  | **HR** | **(95 %CI)** |
| rs2104286 | 0 | 1,272 | 1.0 |  | 0 | 1.0 |  | 893 | 1.0 |  | 0 | 1.0 |  |
|  | 1 | 878 | 1.06 | (0.86 - 1.31) | 1/2 | 1.07 | (0.88 - 1.31) | 574 | 0.96 | (0.75 - 1.22) | 1/2 | 1.00 | (0.79 - 1.27) |
|  | 2 | 147 | 1.12 | (0.72 - 1.75) |  |  |  | 109 | 1.37 | (0.85 - 2.22) |  |  |  |
| rs1678542 | 0 | 978 | 1.0 |  | 0 | 1.0 |  | 673 | 1.0 |  | 0 | 1.0 |  |
|  | 1 | 1,018 | 1.04 | (0.84 - 1.29) | 1/2 | 0.99 | (0.81 - 1.21) | 698 | 1.05 | (0.82 - 1.35) | 1/2 | 0.98 | (0.78 - 1.24) |
|  | 2 | 292 | 0.83 | (0.60 - 1.15) |  |  |  | 199 | 0.79 | (0.53 - 1.16) |  |  |  |
| rs763361 | 0 | 636 | 1.0 |  | 0 | 1.0 |  | 449 | 1.0 |  | 0 | 1.0 |  |
|  | 1 | 1,127 | 0.81 | (0.64 - 1.03) | 1/2 | 0.85 | (0.68 - 1.06) | 756 | 0.84 | (0.64 - 1.11) | 1/2 | 0.88 | (0.68 - 1.14) |
|  | 2 | 538 | 0.93 | (0.71 - 1.22) |  |  |  | 374 | 0.97 | (0.70 - 1.33) |  |  |  |
| rs3087243 | 0 | 705 | 1.0 |  | 0 | 1.0 |  | 487 | 1.0 |  | 0 | 1.0 |  |
|  | 1 | 1,082 | 1.07 | (0.85 - 1.34) | 1/2 | 1.1 | (0.89 - 1.37) | 738 | 0.98 | (0.75 - 1.29) | 1/2 | 1.05 | (0.82 - 1.35) |
|  | 2 | 472 | 1.21 | (0.91 - 1.61) |  |  |  | 327 | 1.24 | (0.89 - 1.71) |  |  |  |
| rs1160542 | 0 | 632 | 1.0 |  | 0 | 1.0 |  | 435 | 1.0 |  | 0 | 1.0 |  |
|  | 1 | 1,151 | 0.99 | (0.79 - 1.25) | 1/2 | 0.96 | (0.77 - 1.20) | 769 | 1.06 | (0.81 - 1.39) | 1/2 | 1.02 | (0.79 - 1.32) |
|  | 2 | 515 | 0.9 | (0.67 - 1.19) |  |  |  | 372 | 0.96 | (0.69 - 1.33) |  |  |  |
| rs6822844 | 0 | 1,579 | 1.0 |  | 0 | 1.0 |  | 1,094 | 1.0 |  | 0 | 1.0 |  |
|  | 1 | 650 | 1.02 | (0.82 - 1.27) | 1/2 | 0.99 | (0.80 - 1.22) | 441 | 0.86 | (0.66 - 1.11) | 1/2 | 0.84 | (0.65 - 1.09) |
|  | 2 | 68 | 0.69 | (0.36 - 1.35) |  |  |  | 40 | 0.68 | (0.30 - 1.53) |  |  |  |
| rs6920220 | 0 | 1,347 | 1.0 |  | 0 | 1.0 |  | 935 | 1.0 |  | 0 | 1.0 |  |
|  | 1 | 819 | 1.02 | (0.83 - 1.26) | 1/2 | 1.04 | (0.85 - 1.27) | 554 | 1.04 | (0.82 - 1.33) | 1/2 | 1.03 | (0.81 - 1.30) |
|  | 2 | 136 | 1.18 | (0.77 - 1.82) |  |  |  | 90 | 0.94 | (0.54 - 1.62) |  |  |  |
| rs231775 | 0 | 910 | 1.0 |  | 0 | 1.0 |  | 636 | 1.0 |  | 0 | 1.0 |  |
|  | 1 | 1,070 | 0.85 | (0.68 - 1.05) | 1/2 | 0.84 | (0.69 - 1.03) | 726 | 0.83 | (0.64 - 1.06) | 1/2 | 0.83 | (0.65 - 1.05) |
|  | 2 | 341 | 0.83 | (0.61 - 1.13) |  |  |  | 232 | 0.82 | (0.57 - 1.18) |  |  |  |
| rs7574865 | 0 | 1,381 | 1.0 |  | 0 | 1.0 |  | 928 | 1.0 |  | 0 | 1.0 |  |
|  | 1 | 821 | 1.06 | (0.86 - 1.31) | 1/2 | 1.04 | (0.85 - 1.28) | 586 | 1.00 | (0.78 - 1.27) | 1/2 | 1.01 | (0.80 - 1.28) |
|  | 2 | 120 | 0.92 | (0.58 - 1.46) |  |  |  | 81 | 1.10 | (0.65 - 1.87) |  |  |  |
| rs4810485 | 0 | 1,332 | 1.0 |  | 0 | 1.0 |  | 928 | 1.0 |  | 0 | 1.0 |  |
|  | 1 | 842 | 1.05 | (0.85 - 1.29) | 1/2 | 1.06 | (0.87 - 1.29) | 570 | 1.01 | (0.79 - 1.29) | 1/2 | 1.01 | (0.80 - 1.28) |
|  | 2 | 138 | 1.12 | (0.72 - 1.76) |  |  |  | 93 | 1.05 | (0.61 - 1.82) |  |  |  |
| rs743777 | 0 | 1,061 | 1.0 |  | 0 | 1.0 |  | 722 | 1.0 |  | 0 | 1.0 |  |
|  | 1 | 1,006 | 1.02 | (0.83 - 1.26) | 1/2 | 1.03 | (0.85 - 1.26) | 690 | 1.06 | (0.83 - 1.35) | 1/2 | 1.03 | (0.81 - 1.29) |
|  | 2 | 256 | 1.08 | (0.77 - 1.52) |  |  |  | 184 | 0.89 | (0.58 - 1.37) |  |  |  |
| rs6897932 | 0 | 1,265 | 1.0 |  | 0 | 1.0 |  | 869 | 1.0 |  | 0 | 1.0 |  |
|  | 1 | 882 | 1.06 | (0.86 - 1.31) | 1/2 | 1.02 | (0.83 - 1.24) | 606 | 1.00 | (0.78 - 1.28) | 1/2 | 0.96 | (0.76 - 1.21) |
|  | 2 | 174 | 0.81 | (0.54 - 1.21) |  |  |  | 120 | 0.75 | (0.47 - 1.21) |  |  |  |
| rs5029937 | 0 | 2,143 | 1.0 |  | 0 | 1.0 |  | 1,471 | 1.0 |  | 0 | 1.0 |  |
|  | 1 | 177 | 1.07 | (0.73 - 1.57) | 1/2 | 1.10 | (0.75 - 1.59) | 122 | 0.85 | (0.52 - 1.38) | 1/2 | 0.88 | (0.54 - 1.42) |
|  | 2 | 3 | 2.88 | (0.40 - 20.68) |  |  |  | 3 | 2.84 | (0.39 - 20.51) |  |  |  |
| rs13207033 | 0 | 1,290 | 1.0 |  | 0 | 1.0 |  | 878 | 1.0 |  | 0 | 1.0 |  |
|  | 1 | 844 | 1.05 | (0.85 - 1.29) | 1/2 | 1.00 | (0.82 - 1.23) | 593 | 1.05 | (0.83 - 1.34) | 1/2 | 1.00 | (0.79 - 1.27) |
|  | 2 | 158 | 0.76 | (0.48 - 1.20) |  |  |  | 105 | 0.71 | (0.40 - 1.25) |  |  |  |

**Supplementary Table 3.** CVD mortality by RA susceptibility SNP loci

| **SNP and no. of copies of risk allele** | | **CVD Mortality as at end of 2007, adjusted by sex** | | | | | | | | | | | |
| --- | --- | --- | --- | --- | --- | --- | --- | --- | --- | --- | --- | --- | --- |
|  |  | **IP cohort** | | | | | | **RA subgroup  (met ACR criteria for RA by end of 2007)** | | | | | |
|  |  | **n** | **HR** | **(95 %CI)** |  | **HR** | **(95 %CI)** | **n** | **HR** | **(95 %CI)** |  | **HR** | **(95 %CI)** |
| rs2104286 | 0 | 1,272 | 1.0 |  | 0 | 1.0 |  | 893 | 1.0 |  | 0 | 1.0 |  |
|  | 1 | 878 | 0.91 | (0.68 - 1.20) | 1/2 | 0.94 | (0.72 - 1.23) | 574 | 0.84 | (0.60 - 1.17) | 1/2 | 0.94 | (0.69 - 1.28) |
|  | 2 | 147 | 1.22 | (0.70 - 2.13) |  |  |  | 109 | 1.75 | (1.00 - 3.09) |  |  |  |
| rs1678542 | 0 | 978 | 1.0 |  | 0 | 1.0 |  | 673 | 1.0 |  | 0 | 1.0 |  |
|  | 1 | 1,018 | 0.87 | (0.66 - 1.16) | 1/2 | 0.87 | (0.67 - 1.14) | 698 | 0.84 | (0.60 - 1.17) | 1/2 | 0.83 | (0.61 - 1.12) |
|  | 2 | 292 | 0.88 | (0.59 - 1.32) |  |  |  | 199 | 0.80 | (0.50 - 1.29) |  |  |  |
| rs763361 | 0 | 636 | 1.0 |  | 0 | 1.0 |  | 449 | 1.0 |  | 0 | 1.0 |  |
|  | 1 | 1,127 | 0.75 | (0.55 - 1.03) | 1/2 | 0.81 | (0.61 - 1.09) | 756 | 0.86 | (0.60 - 1.24) | 1/2 | 0.92 | (0.65 - 1.28) |
|  | 2 | 538 | 0.94 | (0.66 - 1.35) |  |  |  | 374 | 1.04 | (0.68 - 1.58) |  |  |  |
| rs3087243 | 0 | 705 | 1.0 |  | 0 | 1.0 |  | 487 | 1.0 |  | 0 | 1.0 |  |
|  | 1 | 1,082 | 0.99 | (0.73 - 1.33) | 1/2 | 0.99 | (0.75 - 1.31) | 738 | 0.88 | (0.62 - 1.23) | 1/2 | 0.89 | (0.65 - 1.22) |
|  | 2 | 472 | 1.02 | (0.69 - 1.49) |  |  |  | 327 | 0.93 | (0.60 - 1.43) |  |  |  |
| rs1160542 | 0 | 632 | 1.0 |  | 0 | 1.0 |  | 435 | 1.0 |  | 0 | 1.0 |  |
|  | 1 | 1,151 | 1.21 | (0.88 - 1.65) | 1/2 | 1.11 | (0.82 - 1.50) | 769 | 1.26 | (0.88 - 1.79) | 1/2 | 1.10 | (0.79 - 1.54) |
|  | 2 | 515 | 0.9 | (0.61 - 1.34) |  |  |  | 372 | 0.81 | (0.52 - 1.27) |  |  |  |
| rs6822844 | 0 | 1,579 | 1.0 |  | 0 | 1.0 |  | 1,094 | 1.0 |  | 0 | 1.0 |  |
|  | 1 | 650 | 1.08 | (0.82 - 1.44) | 1/2 | 1.04 | (0.79 - 1.37) | 441 | 0.92 | (0.66 - 1.28) | 1/2 | 0.88 | (0.64 - 1.22) |
|  | 2 | 68 | 0.67 | (0.28 - 1.64) |  |  |  | 40 | 0.56 | (0.18 - 1.77) |  |  |  |
| rs6920220 | 0 | 1,347 | 1.0 |  | 0 | 1.0 |  | 935 | 1.0 |  | 0 | 1.0 |  |
|  | 1 | 819 | 0.92 | (0.70 - 1.22) | 1/2 | 0.94 | (0.72 - 1.23) | 554 | 0.91 | (0.66 - 1.26) | 1/2 | 0.91 | (0.67 - 1.24) |
|  | 2 | 136 | 1.06 | (0.58 - 1.91) |  |  |  | 90 | 0.88 | (0.43 - 1.81) |  |  |  |
| rs231775 | 0 | 910 | 1.0 |  | 0 | 1.0 |  | 636 | 1.0 |  | 0 | 1.0 |  |
|  | 1 | 1,070 | 0.97 | (0.73 - 1.29) | 1/2 | 0.96 | (0.73 - 1.25) | 726 | 0.91 | (0.66 - 1.26) | 1/2 | 0.90 | (0.66 - 1.22) |
|  | 2 | 341 | 0.91 | (0.60 - 1.37) |  |  |  | 232 | 0.86 | (0.53 - 1.38) |  |  |  |
| rs7574865 | 0 | 1,381 | 1.0 |  | 0 | 1.0 |  | 928 | 1.0 |  | 0 | 1.0 |  |
|  | 1 | 821 | 0.99 | (0.75 - 1.30) | 1/2 | 0.98 | (0.75 - 1.28) | 586 | 0.90 | (0.66 - 1.24) | 1/2 | 0.91 | (0.67 - 1.23) |
|  | 2 | 120 | 0.93 | (0.52 - 1.69) |  |  |  | 81 | 0.92 | (0.45 - 1.90) |  |  |  |
| rs4810485 | 0 | 1,332 | 1.0 |  | 0 | 1.0 |  | 928 | 1.0 |  | 0 | 1.0 |  |
|  | 1 | 842 | 1.03 | (0.78 - 1.35) | 1/2 | 1.06 | (0.82 - 1.38) | 570 | 0.99 | (0.72 - 1.37) | 1/2 | 1.07 | (0.79 - 1.45) |
|  | 2 | 138 | 1.35 | (0.78 - 2.35) |  |  |  | 93 | 1.71 | (0.96 - 3.06) |  |  |  |
| rs743777 | 0 | 1,061 | 1.0 |  | 0 | 1.0 |  | 722 | 1.0 |  | 0 | 1.0 |  |
|  | 1 | 1,006 | 1.21 | (0.92 - 1.59) | 1/2 | 1.22 | (0.93 - 1.58) | 690 | 1.23 | (0.89 - 1.69) | 1/2 | 1.22 | (0.90 - 1.66) |
|  | 2 | 256 | 1.25 | (0.80 - 1.95) |  |  |  | 184 | 1.21 | (0.72 - 2.03) |  |  |  |
| rs6897932 | 0 | 1,265 | 1.0 |  | 0 | 1.0 |  | 869 | 1.0 |  | 0 | 1.0 |  |
|  | 1 | 882 | 1.07 | (0.81 - 1.41) | 1/2 | 1.01 | (0.78 - 1.32) | 606 | 1.00 | (0.73 - 1.38) | 1/2 | 0.97 | (0.72 - 1.32) |
|  | 2 | 174 | 0.78 | (0.45 - 1.33) |  |  |  | 120 | 0.85 | (0.47 - 1.52) |  |  |  |
| rs5029937 | 0 | 2,143 | 1.0 |  | 0 | 1.0 |  | 1,471 | 1.0 |  | 0 | 1.0 |  |
|  | 1 | 177 | 1.21 | (0.76 - 1.94) | 1/2 | 1.20 | (0.75 - 1.92) | 122 | 0.91 | (0.49 - 1.68) | 1/2 | 0.90 | (0.49 - 1.65) |
|  | 2 | 3 | - | - |  |  |  | 3 | - | - |  |  |  |
| rs13207033 | 0 | 1,290 | 1.0 |  | 0 | 1.0 |  | 878 | 1.0 |  | 0 | 1.0 |  |
|  | 1 | 844 | 1.10 | (0.83 - 1.44) | 1/2 | 1.07 | (0.82 - 1.39) | 593 | 1.00 | (0.73 - 1.37) | 1/2 | 1.00 | (0.74 - 1.35) |
|  | 2 | 158 | 0.94 | (0.54 - 1.64) |  |  |  | 105 | 1.01 | (0.54 - 1.89) |  |  |  |
